# MicroRNA-188 suppresses G_1_/S transition by targeting multiple cyclin/CDK complexes

**DOI:** 10.1186/s12964-014-0066-6

**Published:** 2014-10-11

**Authors:** Jiangbin Wu, Qing Lv, Jie He, Haoxiang Zhang, Xueshuang Mei, Kai Cui, Nunu Huang, Weidong Xie, Naihan Xu, Yaou Zhang

**Affiliations:** School of Life Sciences, Tsinghua University, Beijing, 100084 PR China; Division of Life Science, Key Lab in Healthy Science and Technology, Graduate School at Shenzhen, Tsinghua University, Shenzhen, 518055 PR China; ENT Department, Peking University Shenzhen Hospital, Shenzhen, 518055 PR China

**Keywords:** MiR-188, Cell cycle, G_1_/S transition, CDK, Cyclin, Rb, E2F

## Abstract

**Background:**

Accelerated cell cycle progression is the common feature of most cancers. MiRNAs can act as oncogenes or tumor suppressors by directly modulating cell cycle machinery. It has been shown that miR-188 is upregulated in UVB-irradiated mouse skin and human nasopharyngeal carcinoma CNE cells under hypoxic stress. However, little is known about the function of miR-188 in cell proliferation and growth control.

**Results:**

Overexpression of miR-188 inhibits cell proliferation, tumor colony formation and G_1_/S cell cycle transition in human nasopharyngeal carcinoma CNE cells. Using bioinformatics approach, we identify a series of genes regulating G_1_/S transition as putative miR-188 targets. MiR-188 inhibits both mRNA and protein expression of CCND1, CCND3, CCNE1, CCNA2, CDK4 and CDK2, suppresses Rb phosphorylation and downregulates E2F transcriptional activity. The expression level of miR-188 also inversely correlates with the expression of miR-188 targets in human nasopharyngeal carcinoma (NPC) tissues. Moreover, studies in xenograft mouse model reveal that miR-188 is capable of inhibiting tumor initiation and progression by suppressing target genes expression and Rb phosphorylation.

**Conclusions:**

This study demonstrates that miR-188 exerts anticancer effects, *via* downregulation of multiple G_1_/S related cyclin/CDKs and Rb/E2F signaling pathway.

**Electronic supplementary material:**

The online version of this article (doi:10.1186/s12964-014-0066-6) contains supplementary material, which is available to authorized users.

## Background

MicroRNAs (miRNAs) are a class of small non-coding RNAs that negatively regulate gene expression at the post-transcriptional level [1]. MiRNAs are important regulators of various biological and pathological processes, including cell proliferation, differentiation, apoptosis, metabolism and cancer [2-11]. As “micromanagers” of gene expression, miRNAs often have subtle influence on one single target. A variety of studies indicate that an individual miRNA may perform its function through targeting multiple components in one signaling pathway [9,12,13]. In addition, multiple miRNAs can act on the same signaling pathway or biological process [14-17].

Cell cycle progression is directly driven by a series of heterodimers formed by cyclins and cyclin-dependent kinases (CDKs) [18]. The decision for a cell to enter S phase is tightly controlled by cyclin D/CDK4/6 and cyclin E/CDK2 complexes, followed by cyclin A/CDK2 throughout S phase [18-20]. In mammalian cells, G_1_ to S phase progression is also regulated by the Retinoblastoma protein (Rb). Rb is a tumor suppressor protein that plays a pivotal role in the negative control of cell cycle and in tumor progression [21,22]. The Rb protein inhibits the expression of genes required for entry into S phase by sequestering the E2F family of transcription factors [23,24]. During G_1_ phase of the cell cycle, Rb binds to E2F-DP1 and inhibits downstream transcription. When it is time for a cell to enter S phase, the inactivating phosphorylation of Rb by the G_1_/S CDKs results in the release of Rb from E2F-DP1 and allows for the activation of E2F target genes that are responsible for facilitating G_1_/S transition and S phase progression.

Several lines of evidence indicate that miRNAs are important cell cycle regulators. Some miRNAs regulate cell cycle progression by directly suppressing the expression of cyclin/CDK complexes. For instance, miR-15/16 family induces cell cycle arrest by simultaneously targeting multiple cyclins that regulate G_1_/S transition; these include CCND3, CCNE1 and CDK6 [25,26]. CDK6 and CCND1 are also regulated by miR-34a, which induces a significant G_1_ cell cycle arrest in human lung carcinoma A549 cells [27]. CDK6 is targeted by miR-137 and miR-124a. The expression of both of these miRNAs is epigenetically silenced by hypermethylation in some human cancers [28-30]. Transfection of miR-137 or miR-124a causes G_1_ arrest in glioblastoma multiforme cells [31]. In addition, miR-206 suppresses CCND1 expression and plays a tumor suppressive function in skeletal muscle differentiation [32]. Besides cyclin/CDK complexes, miRNAs also modulate cell proliferation through interacting with critical cell cycle regulators that are involved in G_1_/S and G_2_/M transitions, such as Cip/Kip and INK4a/ARF family members, E2F family transcription factors, Cdc25, Wee1 and p53, etc. [33]. Therefore, better understanding the roles of miRNAs in cell proliferation and cell cycle checkpoints may shed light on cancer pathogenesis and lead to rational development of new types of cancer therapy.

MiR-188, a miRNA located on the X chromosome in humans, was first identified in 2003 [34]. It has been reported that miR-188 is upregulated by the induction of long-term potentiation (LTP) and it serves to fine-tune synaptic plasticity by targeting neuropilin-2 (Nrp-2) expression in the nervous system [35]. Recent studies also show that miR-188 is upregulated in UVB-irradiated mouse skin and human nasopharyngeal carcinoma CNE cells under hypoxic stress [36,37]. However, little is known about the function of miR-188 on cell proliferation and cell cycle regulation. Here, we report that miR-188 is a potent tumor suppressor in human nasopharyngeal carcinoma cells. Overexpression of miR-188 inhibits cell proliferation and G_1_/S transition by directly targeting the expression of multiple cyclin/CDKs complexes, including CCND1, CCND3, CCNE1, CCNA2, CDK2 and CDK4. MiR-188 also suppresses Rb phosphorylation and E2F transcriptional activity. Further study from xenograft mouse model shows that miR-188 has anti-cancer potential through suppressing tumor initiation and progression. Taken together, our data highlight the anti-cancer potential of miR-188 and provide a novel mechanism whereby miR-188 controls cell cycle progression via downregulation of multiple cyclin/CDK complexes involved in G_1_/S transition.

## Results

### Overexpression of miR-188 suppresses cancer cell proliferation

**Figure 1:**
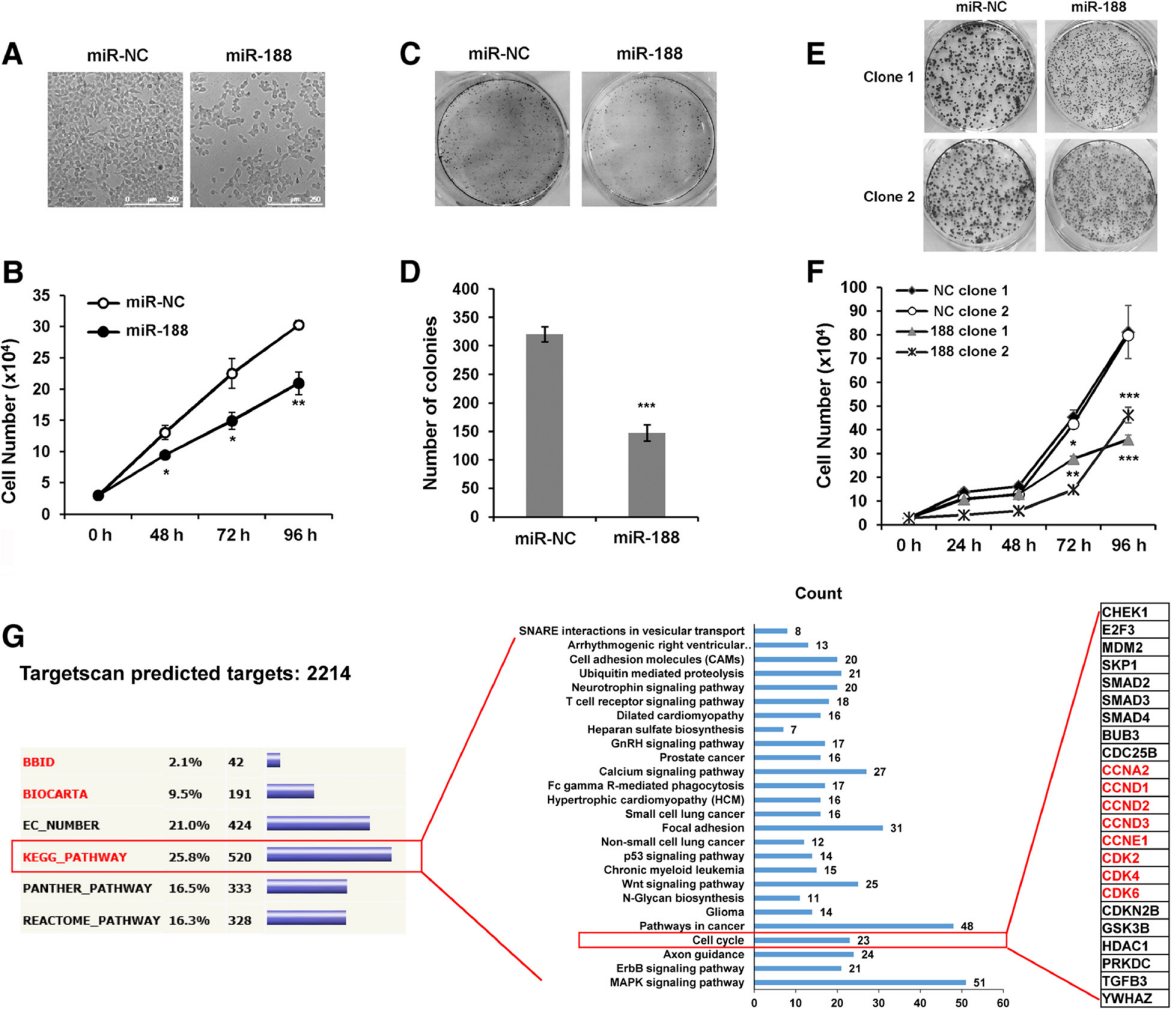
**MiR-188 inhibits cell proliferation and tumor colony formation. (A)** Overexpression of miR-188 inhibits cell growth in CNE cells. CNE cells were transfected with miR-188 or miR-NC for 72 h, the representative phase contrast images were shown. Scar bar: 250 μm. **(B)** Overexpression of miR-188 inhibits cell proliferation. Equal amounts of CNE cells were transfected with miR-188 or miR-NC, cell proliferation was monitored at indicated time points. Data presented are means ± SD from three independent experiments (Student *t* test, *p < 0.05, **p < 0.01). **(C)** Clonogenic assay of CNE cells transfected with miR-NC or miR-188. Cells were cultured for 10 days and stained with Crystal Violet. **(D)** The number of colonies were quantified and calculated as means ± SD of three independent experiments (Student *t* test, ***p < 0.001). **(E)** Clonogenic assay of CNE cells stably expressing miR-NC or miR-188. **(F)** Stable transfection of miR-188 inhibits cell proliferation. Equal amounts of miR-NC or miR-188 stable cell lines were cultured for 4 days, cell proliferation was monitored at indicated time points. Data shown are means ± SD from three independent experiments (Student *t* test, *p < 0.05, **p < 0.01, ***p < 0.001). **(G)** The functional association of miR-188 target genes. DAVID Functional Annotation tool was used for analysis of potential targets predicted from Targetscan and Findtar. A series of cell cycle related genes were potential targets of miR-188.

To characterize the function of miR-188 on cancer cell proliferation, miR-188 mimics or negative control (miR-NC) were transiently transfected into human nasopharyngeal cancer CNE cells. Strikingly, we observed that overexpression of miR-188 attenuated cell proliferation in CNE cells (Figure 1A and B). The effect of miR-188 on cell proliferation was also evaluated using colony formation assay. MiR-188 transfected cells formed significantly less colonies than those transfected with the negative control (Figure 1C and D).

By quantifying the level of miR-188 in CNE cells, we found that the expression of miR-188 increased by nearly 1000 folds after transient transfection of miRNA mimics, which is far beyond the level of endogenous miR-188 (Additional file 1: Figure S1A). To create a scenario closer in resemblance to the normal physiological state, we generated CNE cells that stably expressed miR-188 to ascertain its effect on cell proliferation. We selected two stable clones in which the expression of miR-188 was nearly 4 folds higher than the negative control (Additional file 1: Figure S1B). Although, there was no significant difference among the colonies between each plate, the colony size of cells stably expressed miR-188 was much smaller than that of the control (Figure 1E). Similar to transiently transfected cells, cell proliferation rate was significantly reduced in the miR-188 stably expressed clones (Figure 1F). Our data indicate that miR-188 expressed cells have slower growth and cell proliferate rate.

### MiR-188 targets multiple cyclin/CDKs involved in G1/S transition

To investigate the potential targets of miR-188, we searched the genome using miRNA target prediction algorithms, Targetscan and Findtar. We found that 2214 genes contain potential miR-188 target sites. We then employed DAVID Bioinformatics Resources (http://david.abcc.ncifcrf.gov/home.jsp) for functional annotation clustering and enrichment scoring [38,39]. According to pathway annotation summary, the KEGG (Kyoto Encyclopedia of Genes and Genomes) pathway was classified as containing most of the genes identified by DAVID as potential targets of miR-188 (Figure 1G left panel). The KEGG contains 26 signaling pathways, among these, MAPK signaling pathway (p value: 1.08 × 10^−5^), ErbB signaling pathway (p value: 3.57 × 10^−4^), axon guidance (p value: 5.16 × 10^−3^) and cell cycle (p value: 7.20 × 10^−3^) were the four pathways most significantly associated with miR-188 according to p values (Figure 1G middle panel, Additional file 2: Table S1). Since cell growth and cell proliferation are highly associated with cell cycle regulation, we analyzed 23 of the genes in the cell cycle pathway and identified a series of cyclins and CDKs involved in G_1_/S transition that could be potential miR-188 targets (Figure 1G right panel).

**Figure 2:**
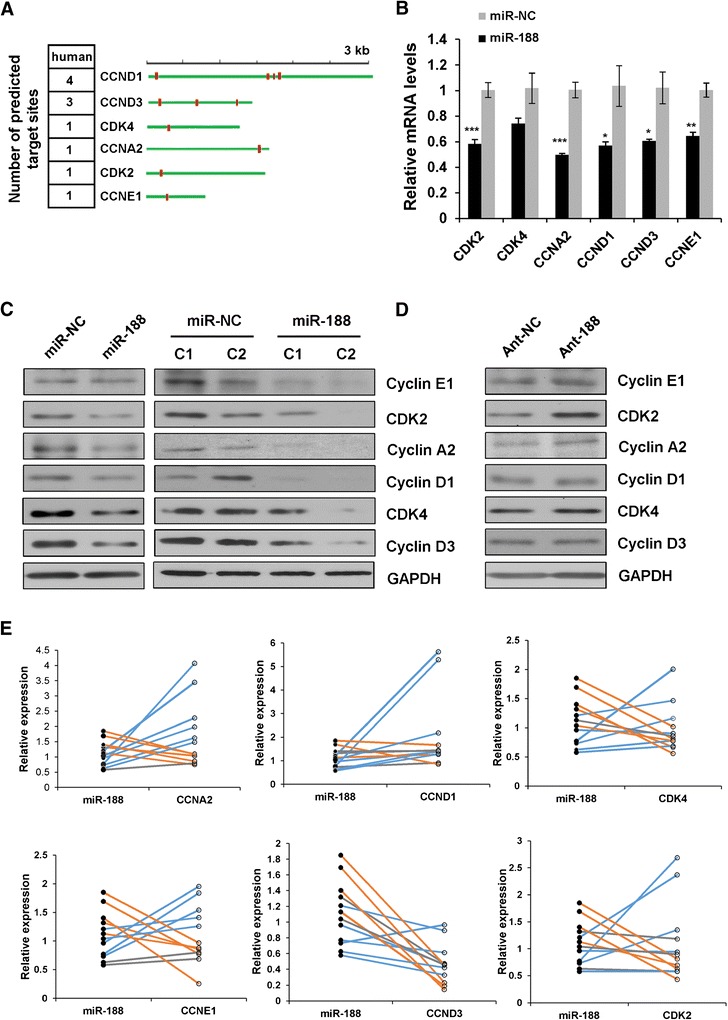
**MiR-188 downregulates multiple genes related to G**_**1**_ **/S transition. (A)** CCND1, CCND3, CDK4, CCNA2, CDK2 and CCNE1 are predicted to be miR-188 target genes. **(B)** qRT-PCR analysis of mRNA expression levels of miR-188 targets in CNE cells transfected with miR-NC or miR-188. Data are presented as means ± SD of three independent experiments (Student *t* test, *p < 0.05, **p < 0.01, ***p < 0.001). **(C)** Overexpression of miR-188 downregulates the protein expression of cyclin E1, CDK2, cyclin A2, cyclin D1, cyclin D3 and CDK4. (C1, stable clone 1; C2, stable clone 2). All western blots were performed at least three times. **(D)** Western blot analysis of cyclin E1, CDK2, cyclin A2, cyclin D1, cyclin D3 and GAPDH in CNE cells transfected with Ant-188 or Ant-NC. All western blots performed at least three times. **(E)** Inverse correlation between miR-188 and its targets in NPC tissues. The relative levels of miR-188, CCND1, CCND3, CDK4, CCNA2, CDK2 and CCNE1 were quantified using qRT-PCR.

Using miRNA target prediction algorithms Findtar, we found that six G_1_/S related genes, including CCND1, CCND3, CDK4, CCNA2, CDK2 and CCNE1 contain miR-188 response elements in their 3′UTRs (Figure 2A). To validate, we overexpressed miR-188 in CNE cells, then harvested cells for mRNA and protein expression level measurements for these six genes. Results from qRT-PCR showed that overexpression of miR-188 resulted in 30-50% reduction in transcript abundance for CCND1, CCNA2, CCND3, CCNE1, CDK2 and CDK4 (Figure 2B). Similarly, western blot experiments demonstrated that the protein levels of these genes were significantly suppressed to various degrees by miR-188 overexpression in both transiently and stably transfected cells (Figure 2C, Additional file 3: Figure S2A, B).

To test whether silencing endogenous miR-188 would affect target gene expression, we transfected cells with miR-188 antisense oligonucleotides (ant-188). Compared to control antisense oligonucleotides (ant-NC), the amounts of CCND1, CCNA2, CCND3, CCNE1, CDK2 and CDK4 proteins increased slightly when endogenous miR-188 expression was silenced (Figure 2D, Additional file 3: Figure S2C). Therefore, these results suggest that miR-188 inhibits cell proliferation, potentially *via* downregulation of multiple cyclin/CDK complexes involved in G_1_/S transition.

### Inverse correlation of miR-188 and its target genes in NPC tissues

To define the clinical relevance of our findings that miR-188 suppressed the expression of G_1_/S related cyclin/CDKs, we showed that miR-188 expression was possibly associated with human nasopharyngeal carcinoma. Namely, we examined the expression of miR-188 and its target genes in NPC tissues using qRT-PCR, and an inverse correlation between miR-188 and CCND1, CCNA2, CCND3, CCNE1, CDK2 or CDK4 expression was identified in patient samples (Figure 2E). Thus, the *in vitro* and *in vivo* results further demonstrate that miR-188 targets the expression of multiple G_1_/S related cyclin/CDKs.

### MiR-188 delays G1/S cell cycle progression

**Figure 3:**
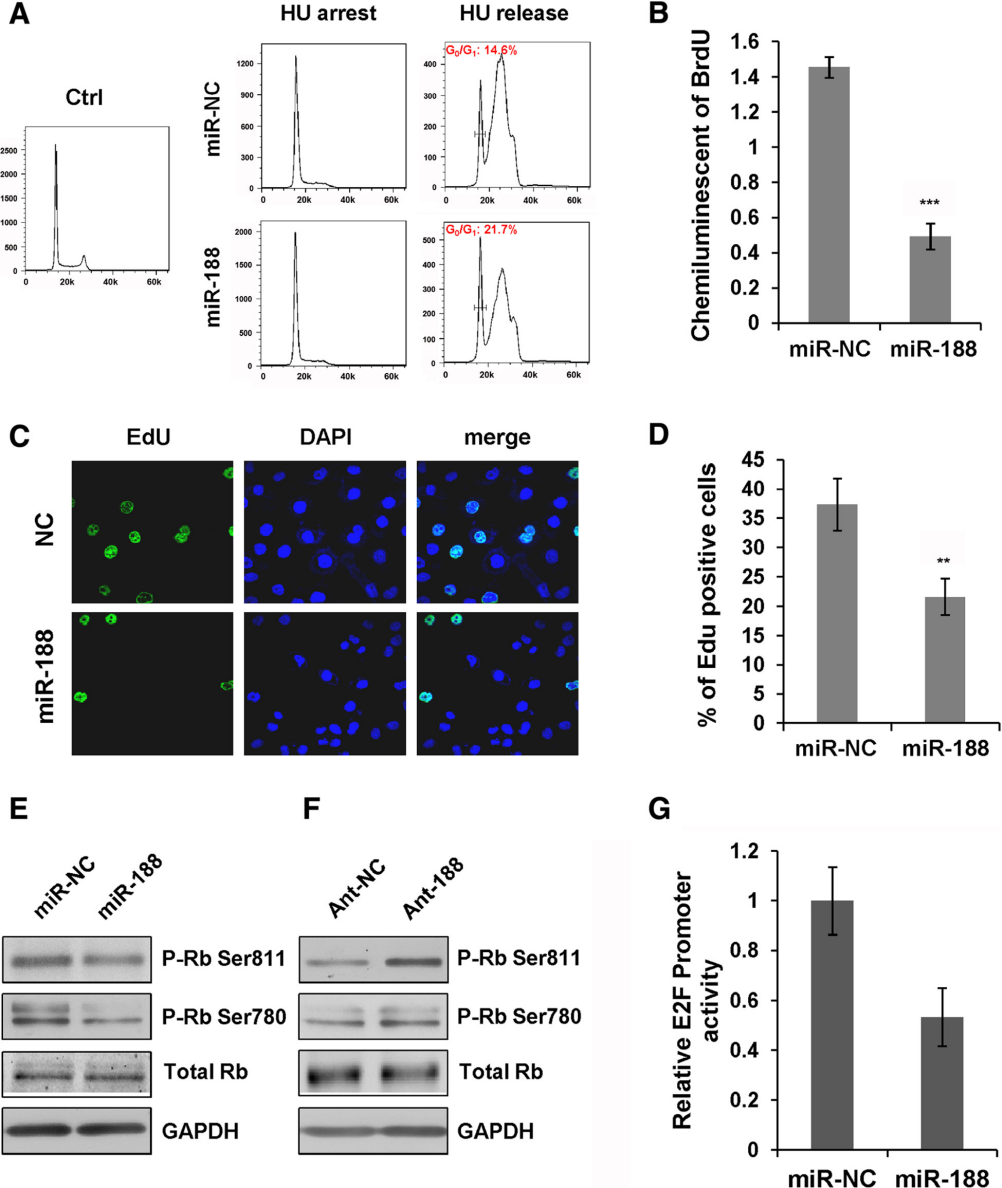
**MiR-188 arrests cell cycle at G**_**1**_ **/S transition through negative regulation of Rb-E2F axis. (A)** CNE cells transfected with miR-188 or miR-NC were synchronized at G1/S boundary by hydroxyurea treatment. Cells were released from hydroxyurea block for 6 h, fixed and stained with propidium iodide (PI) for flow cytometry analysis. **(B)** miR-188 inhibits BrdU incorporation. BrdU Elisa Assay of CNE cells released from hydroxyurea block for 4 h. The BrdU incorporation was quantified by measuring the chemiluminescence. Data shown as means ± SD from three independent experiments (Student *t* test, ***p < 0.001). **(C)** Confocal immunofluorescence analysis of CNE cells stained with EdU (green) and DAPI (blue). Scar bar: 10 μm. **(D)** Quantification of EdU positive cells in CNE cells transfected with miR-188 or miR-NC released from hydroxyurea block for 2 h. Data are presented as means ± SD from three independent experiments (Student *t* test, **p < 0.01). **(E)** Immunoblot analysis of phosphorylated and total Rb expression in CNE cells transfected with miR-NC, miR-188, **(F)** Ant-NC or Ant-188 respectively. GAPDH were used as internal control. **(G)** E2F promoter activity was determined directly by use of a pE2F-TA-Luc luciferase reporter plasmid which contains four E2F binding sites upstream of TA promoter. Data shown as means ± SD of three independent experiments (Student *t* test, *p < 0.05).

Having identified the potential targets of miR-188, we then wanted to determine the role of miR-188 on cell cycle progression, especially on G_1_/S transition. CNE cells transfected with miR-188 or miR-NC were synchronized at G_1_/S boundary by treatment with hydroxyurea. At 6 hours after release from hydroxyurea, the vast majority of control cells were in S and G_2_/M phase (Figure 3A). The G_1_ cell population was bigger in miR-188 transfected CNE cells (21.7 ± 1.38%) than that in control cells (14.6 ± 0.95%) (Figure 3A). Similar results were obtained from miR-188 stably overexpressed cells (Additional file 4: Figure S3A).

One feature of G_1_/S transition is the beginning of genomic DNA synthesis. Chemicals, such as BrdU or EdU, can be inserted into newly synthesized DNA to allow for visualization of the chromosomes. A precise evaluation of cell proliferation can be performed by measuring BrdU or EdU incorporation in proliferating cells during DNA synthesis. First, we performed cell proliferation ELISA BrdU assay. Namely, CNE cells were synchronized at G_1_ phase by treatment with hydroxyurea. Subsequently, they were released and labeled with BrdU for 2 hours. The incorporation of BrdU was then determined by measuring chemiluminescence. There was a significant reduction of BrdU incorporation in miR-188 transfected cells (Figure 3B). This observation was confirmed by EdU image assay. The incorporation of EdU in miR-188 transfected cells was significantly less than that of control cells (Figure 3C and D). Together, these results demonstrate that miR-188 suppresses cell proliferation mainly by interrupting G_1_/S cell cycle progression.

### MiR-188 suppresses Rb phosphorylation and E2F transcriptional activity

E2F activity is crucial for G_1_/S transition and DNA replication in mammalian cells. The tumor suppressor Rb is the primary negative regulator of E2F. Disruption of Rb/E2F interaction is achieved through CDK-mediated phosphorylation of Rb. The initial phosphorylation is performed by Cyclin D/CDK4/CDK6 and followed by additional phosphorylation by Cyclin E/CDK2. Since we have shown that miR-188 downregulates the expression of Cyclin D/CDK4 and Cyclin E/CDK2 complexes, we wanted to ask whether overexpression of miR-188 would have an impact on Rb phosphorylation. The phosphorylation status of Rb at ser780 and ser811 was detected using western blot. We found that miR-188 suppressed CDK-mediated Rb phosphorylation since silencing endogenous miR-188 with ant-188 increased the amount of Rb phosophorylation while miR-188 transfected cells showed less Rb phosphorylation (Figure 3E and F, Additional file 4: Figure S3B). Similarly, Rb phosphorylation at S811 and S780 residues were significantly reduced in CNE cells stably expressing miR-188 (Additional file 4: Figure S3C, D). These results indicate that miR-188 also plays an important role on Rb phosphorylation.

E2F acts as a transcription factor in the nucleus and activates down-stream gene expression to drive cell cycle progression. A reduction of Rb phosphorylation would affect its dissociation from E2F and therefore, affect E2F activation. Thus, we speculated that overexpression of miR-188 would lead to a downregulation of E2F transcriptional activity. Using a pE2F-TA-Luc plasmid that contains four copies of E2F binding elements, we showed that enforced expression of miR-188 remarkably decreased E2F transcriptional activity, as determined by reduced luciferase activity (Figure 3G). These results suggest that miR-188 downregulates CDK-mediated Rb phosphorylation and subsequent activation of E2F.

### MiR-188 directly binds to the 3′UTRs of target genes

**Figure 4:**
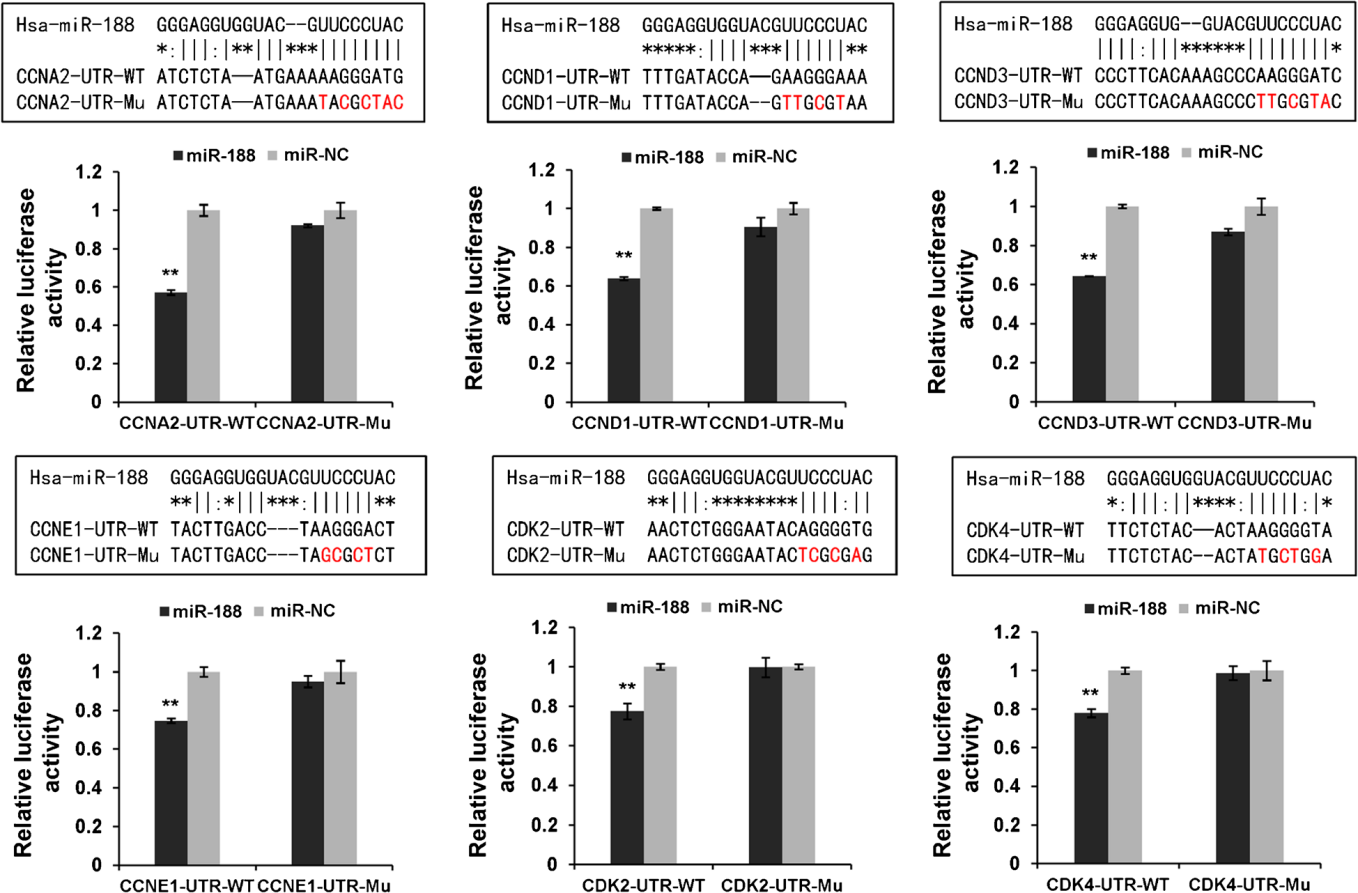
**MiR-188 regulates target genes expression through direct binding to their 3′UTRs.** Sequences of miR-188 and predicted miR-188-binding sites at CCNA2, CCND1, CCND3, CCNE1, CDK2 and CDK4 3′UTRs. The mutant sequences (Mu) are identical to the wild-type (WT) except the mutated nucleotides are shown in red. Luciferase vectors were generated by inserting WT or Mu 3′UTRs of target genes into pmirGLO plasmid. The reporter vectors were co-transfected with miR-188 or miR-NC. Relative luciferase activity was determined at 24 h after transfection. Results shown are means ± SD of three independent experiments (Student *t* test, **p < 0.01).

To determine whether miR-188 indeed target CCND1, CCND3, CCNA2, CCNE1, CDK2 and CDK4, we employed luciferase reporter assay. The potential miR-188 binding sequences within the 3′UTRs of target genes were predicted by FindTar (Figure 4). The 3′UTR fragments of target genes containing wild-type (WT) or seed region mutated (Mu) of miR-188 binding sites were inserted into a dual-luciferase reporter vector. When introduced into cells, the wild-type 3′UTR reporters of CCND1, CCND3, CCNA2, CCNE1, CDK2 and CDK4 exhibited a significant reduction of luciferase activity in the miR-188 transfected cells as compared to control. In contrast, the reporter vectors carrying seed mutated 3′UTR fragments abrogated the inhibitory effect of miR-188 on luciferase activity (Figure 4). Therefore, we concluded that miR-188 inhibits target-gene expression through direct interaction with their 3′UTRs.

### MiR-188 suppresses tumor formation and progression in nude mice

**Figure 5:**
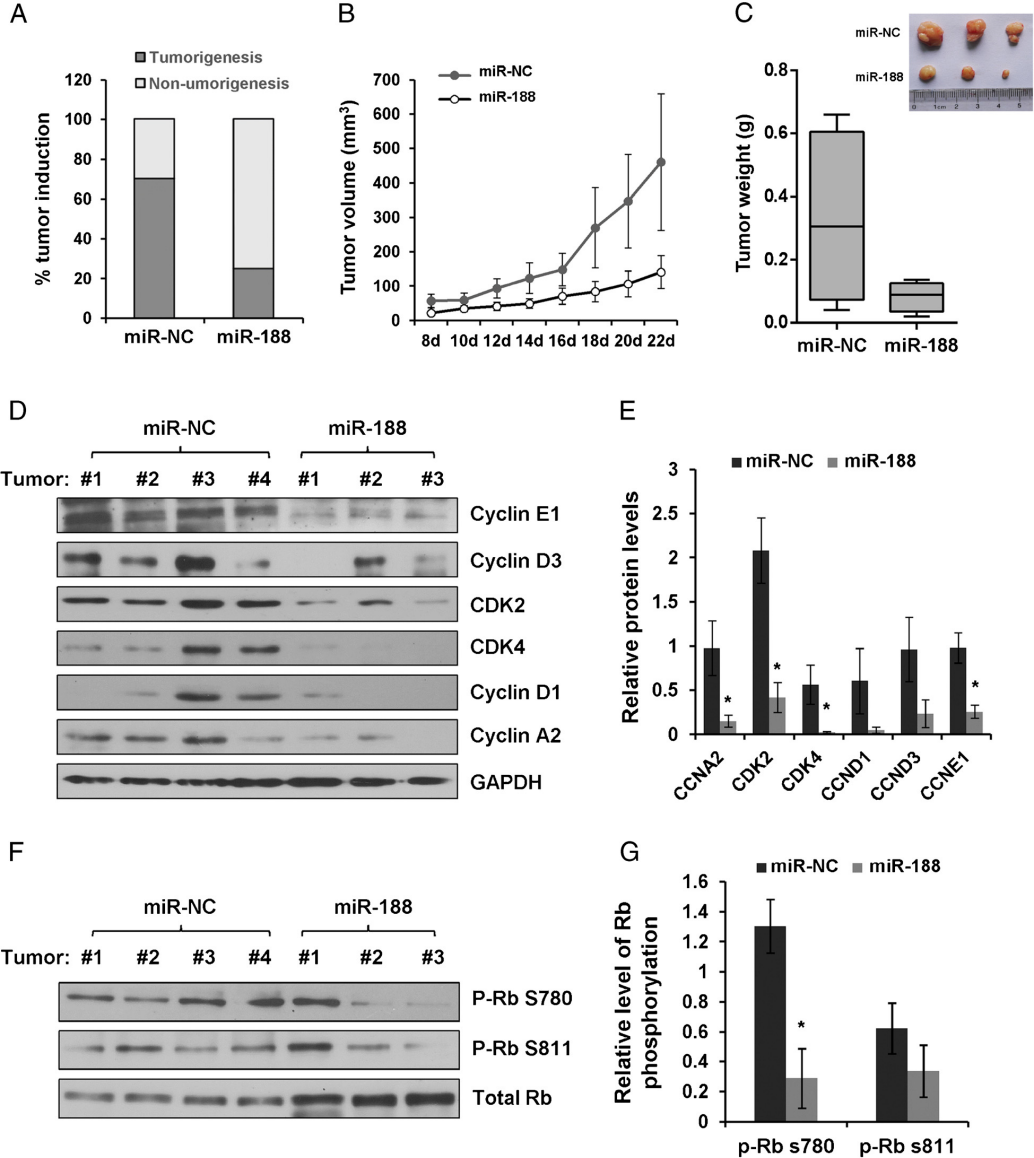
**MiR-188 suppresses tumor initiation and progression** ***in vivo*** **. (A)** CNE cells stably expressing miR-188 or miR-NC were injected into nude mice. MiR-188 (n = 12) suppressed tumorigenesis compared with miR-NC (n = 10). **(B)** miR-188 inhibits tumor growth *in vivo*. Tumor volumes were calculated by the length and width measured by vernier calipers every 2 days. **(C)** The weight of tumors from mice from **(B)**. miR-NC (mean: 327.9 mg; range: 39.6 to 659.4 mg); miR-188 (mean: 83.2 mg; range: 19.5 to 136.6 mg). **(D)** Immunoblot analysis of cyclin D1, cyclin D3, cyclin E1, cyclin A2, CDK2, CDK4 and GAPDH in tumors treated with miR-NC or miR-188. **(E)** Relative protein expression was quantified by densitometric analysis, GAPDH was used as internal control (Student t test, *p < 0.05). **(F)** Immunoblot analysis of phosphor-Rb Ser811, phosphor-Rb Ser780 and total Rb in tumors treated with miR-NC or miR-188. **(G)** Relative Rb phosphorylation was quantified by densitometric analysis, total Rb was used as internal control (Student t test, *p < 0.05).

In order to determine the anti-cancer potential of miR-188 *in vivo*, nude mice were injected with CNE cells that stably expressed miR-188 or miR-NC. Strikingly, tumor induction was dramatically prevented in miR-188 injected mice (Figure 5A). Tumor mass and tumor volume were also significantly lower in mice injected with miR-188 (Figure 5B and C). We then investigated the expression of miR-188 targets in tumor tissues using western blot. The protein levels of Cyclin E1, Cyclin D3, Cyclin D1,Cyclin A2, CDK2 and CDK4 were significantly decreased in tumor tissues harvested from miR-188 injected mice (Figure 5D and E). Compared with tumor tissues from control mice, an obvious reduction of Rb S811 and S780 phosphorylation was also detected in miR-188 injected mice (Figure 5F and G). These results provide strong evidence that miR-188 suppresses tumor initiation and progression *in vivo*, potentially through downregulation of multiple genes involved in G_1_/S transition.

## Discussion

**Figure 6:**
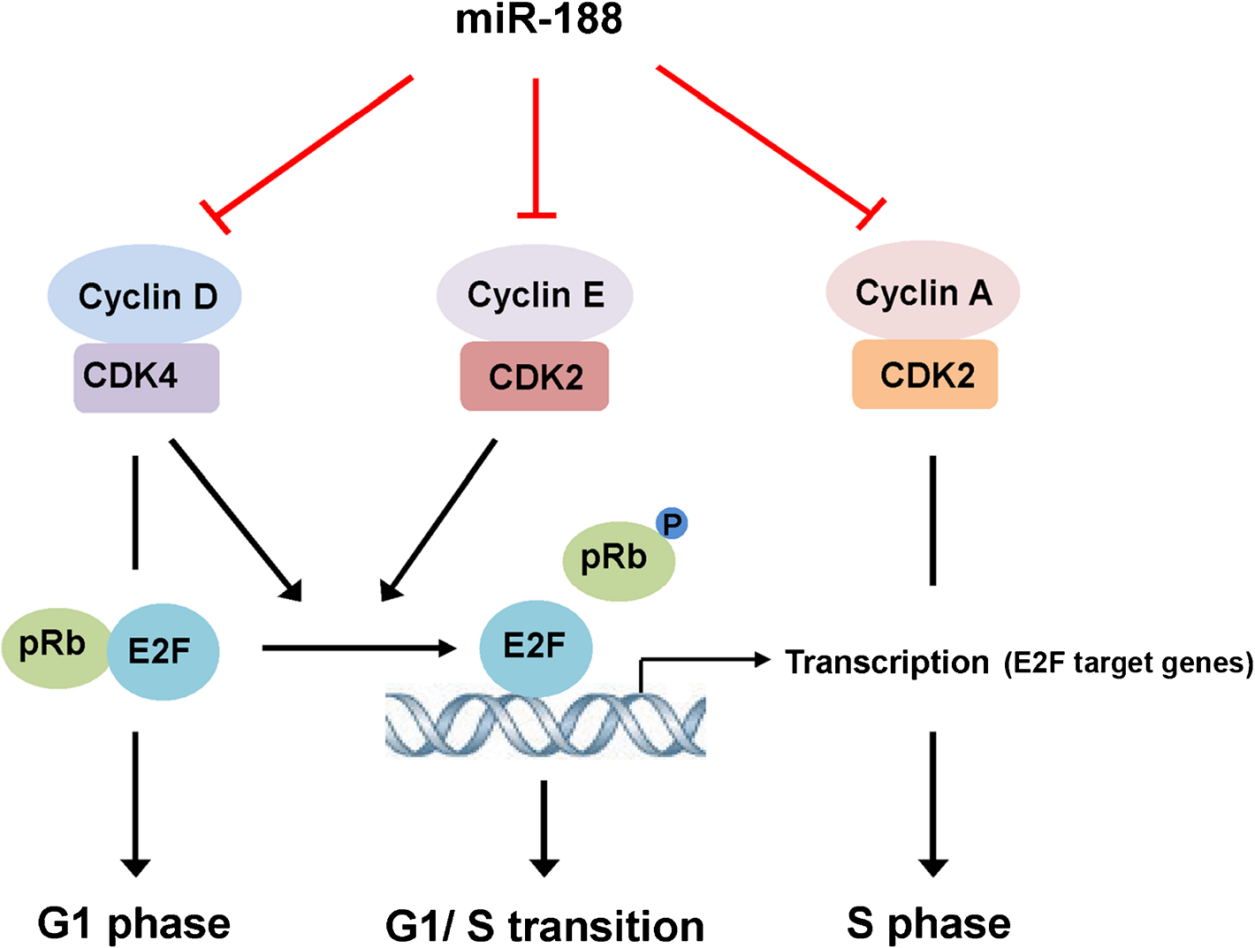
**MiR-188 targets multiple genes involved in G**_**1**_ **/S cell cycle transition.** MiR-188 suppresses cyclin D/CDK4, cyclin E/CDK2 and cyclin A/CDK2 activity by directly targeting their 3′UTRs. MiR-188 mediated downregulation of G_1_/S CDKs suppresses Rb phosphorylation and E2F transcriptional activity, and finally leads to G_1_/S cell cycle arrest and growth inhibition.

Being capable of affecting multiple targets is one of the most important feature of miRNAs. One miRNA can regulate several targets in a signaling pathway, alternatively, several miRNAs can also converge on a single target [13,14,26]. Co-regulation of a group of functionally related genes to provoke detectable functional changes is a common mode of miRNA-mediated gene regulation. In this study, we demonstrate that miR-188 exerts anti-cancer potential *via* downregulating multiple G_1_/S related genes, including CCND1, CCND3, CCNE1, CCNA2, CDK2 and CDK4. By suppressing the activation of G_1_/S related Cyclin/CDKs, miR-188 suppresses Rb phosphorylation and subsequent activation of E2F transcription factor, which leads to G_1_/S cell cycle arrest and growth inhibition in cancer cells (Figure 6).

Cell cycle is driven by the alternative expression or degradation of different cyclins which exert their function by binding to different CDKs [19,40]. There are two main groups of cyclins, G_1_/S Cyclins are essential for the control of cell cycle at the G_1_/S transition, G_2_/M Cyclins are essential for the control of cell cycle at G_2_/M transition and mitosis. Cyclin D is the first cyclin activated by binding to CDK4/6 in the cell cycle progression. The active Cyclin D/CDK4/6 complex phosphorylates Rb protein [41]. The phosphorylated Rb dissociates from Rb-E2F complex and activates E2F transcription factor. E2F drives the expression of multiple genes that are involved in regulating cell cycle progression, such as CCNE, CCNA, DHFR, MYB and DNA polymerase, etc. [42]. Cyclin E/CDK2 complex pushes cell cycle through G_1_/S transition and Cyclin A/CDK2 complex drives cells through S phase. A fundamental aspect of cancer is the dysregulation of cell cycle control. Alterations in the machinery that controls G_1_/S transition are frequently observed in many types of human cancers [43-45]. For instance, amplification of CCND1 gene or overexpression of Cyclin D1 protein has been found in a wide spectrum of human cancers [46,47]. CCND2 and CCND3 genes and their encoded proteins are also overexpressed in many cancer cases in humans [47-51]. Hyperactivation of CDK4 or CDK6 is also found in several human malignancies, which can be achieved through deregulated expression of D-type Cyclins, or loss of CDK inhibitor, p16INK4a [47,52-54]. Due to their critical role in cell cycle progression and tumorigenesis, the Cyclin/CDKs have been proposed as attractive therapeutic targets for anti-cancer treatment.

Accelerated cell cycle progression is the common feature of most cancers. Several lines of evidence implicate miRNAs as oncogenes or tumor suppressors through directly modulating cell cycle machinery [55]. For instance, the oncogenic miR-17/92 and miR-221/222 clusters can downregulate CDK inhibitors and Rb family members, leading to the activation of Cyclin/CDK complexes and cell cycle progression [56-59]. On the other hand, tumor suppressive miRNAs, such as let-7 and miR-15 families, downregulate a wide spectrum of positive regulators of the cell cycle machinery [55]. Our study provides strong evidence supporting miR-188 may function as a tumor suppressor and it is a potential candidate for anti-cancer therapy.

## Conclusions

Our data demonstrate a novel mechanism of miR-188 control on cell proliferation and cell cycle progression. Through targeting multiple cyclin/CDK complexes, miR-188 blocks G_1_/S transition, suppresses Rb phosphorylation and E2F transcriptional activity. The expression of miR-188 also inversely correlates with expression of its targets in NPC tissues. Moreover, miR-188 is capable of inhibiting tumor initiation and progression in xenograft mouse model, indicating that miR-188 may have anti-cancer potential in human nasopharyngeal cancer.

## Materials and methods

### Cell culture and transfection

CNE cell line was purchased from Kunming Cell Bank (China) and cultured in Dulbecco’s modified Eagle’s medium (Gibco/Invitrogen, 12800–017) supplemented with 10% fetal bovine serum (PAA, A15-101) at 37°C in a humidified 5% CO_2_ incubator. Cells were transfected with siRNA or miRNAs duplexes using Lipofectaime 2000 (Invitrogen Corp., Carlsbad, CA, USA) according to manufacturer’s instructions. MiR-188 mimics and miRNA inhibitors were synthesized and purified by GenePharma Co. (Shanghai, China).

### miRNA and mRNA expression

Total RNA from cultured cell were extracted with RNAiso plus (Takara, 9108) following the manufacture’s protocol. For miRNA expression assay, total RNA was reversely transcribed using Taqman MicroRNA Reverse Transcription Kit (Life Technologies, 4366597), then miRNA real-time PCR was performed using Taqman MicroRNA Assay Kit (Life Technologies). For mRNA expression assay, total RNA was reversely transcribed using M-MLV Reverse Transcription System (Takara) and SYBE Green PCR master mix (Toyobo, QPK-201) was purchased for mRNA real-time PCR. All quantitative real-time PCR were performed on an ABI 7300 Real Time System, RNU6 or GAPDH was used as internal control for miRNA and mRNA assay respectively. Relative gene expression was calculated by mean of relative quantification (2^-ΔΔCt^) as previously described [60]. The specific real-time PCR primers for each gene were listed in Table S2 (Additional file 5: Table S2).

### MiR-188 stable cell line

Plasmid carrying GFP and miR-188 hairpin was purchased from GenePharma Co. (Shanghai, China). CNE cells were transiently transfected with miR-188 plasmid, followed by Blasticidin S (YEASEN, 60218ES10) selection at final concentration of 20 ug/ml. Stable clones were obtained based on GFP expression.

### Colony formation and cell proliferation assays

CNE cells were plated at a concentration of 3 × 10^3^ cells per well in 6-well plate and transfected with miR-188 or miR-NC every three day after cells adhesion. MiR-NC or miR-188 stable cells were plated at 4 × 10^3^ cells per well in 6-well plate. After culturing for 10 days, cells were fixed with cold methanol and stained with 1% crystal violet. The number of colonies was counted using Gel-Pro analyzer software. For cell growth assay, cells were plated at a concentration of 2 × 10^4^ cells per well in 24-well plate and transfected with miR-NC or miR-188, the number of viable cells was determined by the trypan-blue exclusion assay.

### Bioinformatics analysis

In order to figure out the potential targets of miR-188, we used Targetscan [61] (http://www.targetscan.org/) and Findtar [62] (http://bio.sz.tsinghua.edu.cn/) algorithm to search human genome based on NCBI mRNA database (http://www.ncbi.nlm.nih.gov/). All predicted targets were uploaded to DAVID Bioinformatics Resources Functional Annotation Tool [38,39] (http://david.abcc.ncifcrf.gov/home.jsp) for pathway annotation and functional annotation clustering.

### Western blotting

Cells were lysed in ice-cold whole cell lysis buffer(50 mM Tris–HCl, pH 8.0, 4 M urea and 1% Triton X-100) supplemented with complete protease inhibitor Cocktail (Roche Diagnostics, 04693132001). Whole cell lysates with equal protein were resolved by SDS-PAGE and transferred to nitrocellulose membrane. After blocking with 5% milk in Tris-buffered saline plus 0.02% Tween-20 (TBST), membranes were incubated with the following antibodies: cyclin D1 (Epitomics, 1677–1), cyclin D3 (Epitomics, 1846–1), E2F1 (Cell Signaling, 3742), cyclin E (Santa Cruz, sc-481), cyclin A (Santa Cruz, sc-596), CDK2 (Cell Signaling, 2546), CDK4 (Cell Signaling, 2906); phosphor-Rb (Ser 780) (Cell Signaling, 3590), phosphor-Rb (Ser-811) (ABclonal Biotechnology, AP0089), total Rb (Cell Signaling, 9309) and GAPDH (Proteintech, 10494-1-AP). Membranes were then incubated with horseradish peroxidase-coupled specific secondary anti-mouse (KPL, 074–1806) or anti-rabbit antibodies (KPL, 474–1506). Protein bands were visualized using ECL blotting detection reagents (KPL, 54-61-00).

### Clinical specimens

All nasopharyngeal carcinoma tissues were collected from tumor resection in the Peking University Shenzhen Hospital (Shenzhen, China). Informed consent was obtained from each patient and this study was approved by the local ethics committee.

### Luciferase activity assay

The 3′ UTRs of CCND1, CCND3, CDK4, CCNA2, CDK2 and CCNE1 containing predicted targets of miR-188 and their corresponding mutants were amplified from reverse transcribed cDNA and cloned to pmirGLO Dual-Luciferase reporter vector (Promega, E1330). The primer sequences for the wild-type (WT) and mutated (Mu) constructs were listed in Table S3 (Additional file 5: Table S3). For luciferase activity assay, WT or Mu constructs were cotransfected with miR-NC or miR-188 into HeLa cell using Lipofectamine 2000. Firefly and Renilla luciferase activity were measured by Dual-Luciferase Reporter Assay System (Promega, E1960). Relative luciferase activity was calculated by normalization the ratio of firefly and Renilla luciferase to that of negative control transfected cells. For E2F activity assay, 200 ng of E2F reporter vector pE2F-TA-Luc (Clontech, 63914) was cotransfected with miR-NC or miR-188 into CNE cells [63]. Luciferase activity was determined at 24 h after transfection.

### Cell synchronization and flow cytometry

For synchronization, cells transfected with miR-NC or miR-188 were treated with 2 mM hydroxyurea (Sigma-Aldrich, H8627) for 16 h to block cell at G1 phase. The synchronized cells were released by washing the cells 3 times with pre-warmed PBS. At 6 h after release, the cells were fixed in 70% ethanol in PBS overnight. Cells were then stained with 10 μg/ml Propidium Iodide (Sigma-Aldrich, P4170) for DNA content analysis by use of a BD Influx™ Flow Cytometer (BD Biosciences).

### BrdU Elisa assay

BrdU elisa assay was performed by use of a Cell proliferation Brdu Elisa Assay kit (Roche, 11647229001). CNE cells transfected with indicated small RNAs were synchronized at G1 phase by hydroxyurea treatment. Cells were released and cultured with 10 μM BrdU for 2 h. After removing labeling medium, cells were fixed and incubated with anti-BrdU-POD working solution for 90 min. Cells were washed 3 times with washing solution and added substrate solution and incubated for 3–10 min at room temperature. The incorporation of BrdU during DNA synthesis in proliferating cells was determined by measuring the light emission of the samples in a microplate luminometer.

### EdU imaging assay

CNE cells transfected with indicated small RNAs were synchronized at G1 phase by hydroxyurea treatment. Cells were released and incubated with 25 μM EdU and subsequently stained using a Click-iT EdU Alexa Fluor 488 Imaging kit (Life Technologies, C10337). Images were taken with an Olympus FV1000 confocal microscope (Olympus, Tokyo, Japan). The percentage of cells labeled with EdU was quantified using Image J software.

### Tumor formation assay

Athymic nude mice aged 4 weeks were purchased from the Experimental Animal Center of Guang Zhou University of Chinese Medicine (Guang Zhou, China) and kept in pathogen-free animal facilities for 2 weeks at Tsinghua University Shenzhen Graduate School. All experimental procedures involving animals were approved by the Institutional Laboratory Animal Care and Use Committee of Experimental Animal Center of Guang Zhou University of Chinese Medicine. Approximately 5 × 10^5^ viable CNE cells stably expressing miR-NC or miR-188 were injected subcutaneously into the dorsal flank of nude mice. Three weeks after tumor implantation, the mice were sacrificed and the tumor incidence and tumor weight of each animal was analyzed. The expression of miR-188 target genes of each tumor was detected by western blot.

### Statistical analysis

Data presented as bar graphs were the means ± S.D. of at least three independent experiments. The student’s *t* test was used to evaluate the significant difference between 2 groups of data. A *P* value less than 0.05 was considered as statistically significant.

## Additional files

Additional file 1: Figure S1. The expression of miR-188 in CNE cells. (A) The relative expression level of miR-188 in CNE cells transiently transfected with miR-188 or miR-NC. (B) The relative expression level of miR-188 in CNE cells stably expressing miR-188 or miR-NC (C1, clone 1; C2, clone 2). Additional file 2: Table S1. DAVID annotation categories the most enrichment KEGG pathway. MAPK signaling pathway, ErbB signaling pathway, Axon guidance and Cell cycle were the first four pathways according to their *p* values. Additional file 3: Figure S2. Densitometric analysis of miR-188 target genes expression. (A) Relative protein levels of cyclin E1, CDK2, CDK4, cyclin A2, cyclin D1 and cyclin D3 in CNE cells transiently transfected with miR-NC or miR-188. (B) Relative protein levels of miR-188 targets in CNE cells stably expressing miR-NC or miR-188 (C1, clone 1; C2, clone 2). (C) Relative protein levels of miR-188 target genes in CNE cells transfected with Ant-NC or Ant-199. GAPDH was used as internal control. Student *t* test, **p* < 0.05, ***p* < 0.01, ****p* < 0.001. Additional file 4: Figure S3. Stable expression of miR-188 inhibits G_1_/S transition and Rb phosphorylation. (A) Flow Cytometry analysis of CNE cells stably expressing miR-NC or miR-188 released from hydroxyurea for 6 h. (B) Relative levels of Rb phosphorylation were quantified by densitometric analysis. Total Rb was used as internal control. Student *t* test, ** p < 0.01, ***p < 0.001. (C) Immunoblot analysis of phosphor-Rb S811, phosphor-Rb S780, total Rb and GAPDH in CNE cells stably expressing miR-NC or miR-188. (D) Relative levels of Rb phosphorylation in (C) were quantified by densitometric analysis, total Rb was used as internal control. Student *t* test, *p < 0.05, ** p < 0.01. Additional file 5: Table S2 and Table S3. Primers used for qRT-PCR (Table S2) and luciferase activity assay (Table S3).

## Abbreviations

CCND1: Cyclin D1
CCND3: Cyclin D3
CDK: Cyclin dependent kinase
CCNA2: Cyclin A2
CCNE1: Cyclin E1
Rb: Retinoblastoma protein
DHFR: Dihydrofolate reductase
ELISA: Enzyme-linked immunosorbent assay
qRT-PCR: Quantitative real-time PCR
HU: Hydroxyurea
EdU: 5-Ethynyl-2′-deoxyuridine
BrdU: 5-bromo-2′-deoxyuridine
UTR: Untranslated region
