# Supplementary material for: MicroRNA-188 suppresses G1/S transition by targeting multiple cyclin/CDK complexes
Source: Cell Commun Signal. 2014 Oct 11;12:66. doi: 10.1186/s12964-014-0066-6 (PMC4200121; doi:10.1186/s12964-014-0066-6)
Supplement: Additional file 1: Figure S1. — The expression of miR-188 in CNE cells. (A) The relative expression level of miR-188 in CNE cells transiently transfected with miR-188 or miR-NC. (B) The relative expression level of miR-188 in CNE cells stably expressing miR-188 or miR-NC (C1, clone 1; C2, clone 2). [file 12964_2014_66_MOESM1_ESM.pptx]

## Slide 1
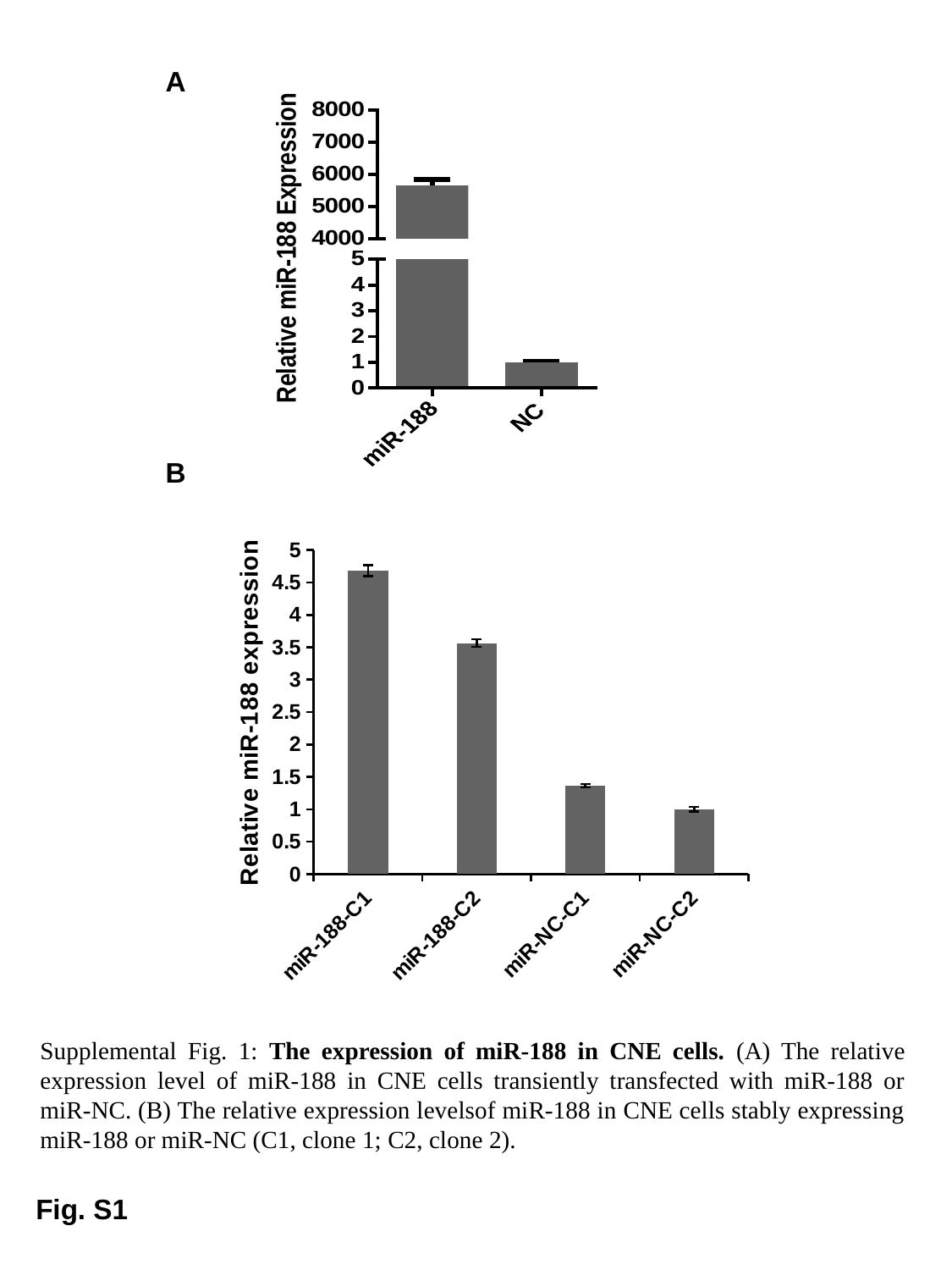

A
B
### Chart
| Category | mir-188 |
|---|---|
| miR-188-C1 | 4.6830760315777935 |
| miR-188-C2 | 3.565583538832865 |
| miR-NC-C1 | 1.363908296322605 |
| miR-NC-C2 | 1.0014846939305542 |Supplemental Fig. 1: The expression of miR-188 in CNE cells. (A) The relative expression level of miR-188 in CNE cells transiently transfected with miR-188 or miR-NC. (B) The relative expression levelsof miR-188 in CNE cells stably expressing miR-188 or miR-NC (C1, clone 1; C2, clone 2).
Fig. S1
