# Supplementary material for: MicroRNA-188 suppresses G1/S transition by targeting multiple cyclin/CDK complexes
Source: Cell Commun Signal. 2014 Oct 11;12:66. doi: 10.1186/s12964-014-0066-6 (PMC4200121; doi:10.1186/s12964-014-0066-6)
Supplement: Additional file 3: Figure S2. — Densitometric analysis of miR-188 target genes expression. (A) Relative protein levels of cyclin E1, CDK2, CDK4, cyclin A2, cyclin D1 and cyclin D3 in CNE cells transiently transfected with miR-NC or miR-188. (B) Relative protein levels of miR-188 targets in CNE cells stably expressing miR-NC or miR-188 (C1, clone 1; C2, clone 2). (C) Relative protein levels of miR-188 target genes in CNE cells transfected with Ant-NC or Ant-199. GAPDH was used as internal control. Student t test, *p < 0.05, **p < 0.01, ***p < 0.001. [file 12964_2014_66_MOESM3_ESM.pptx]

## Slide 1
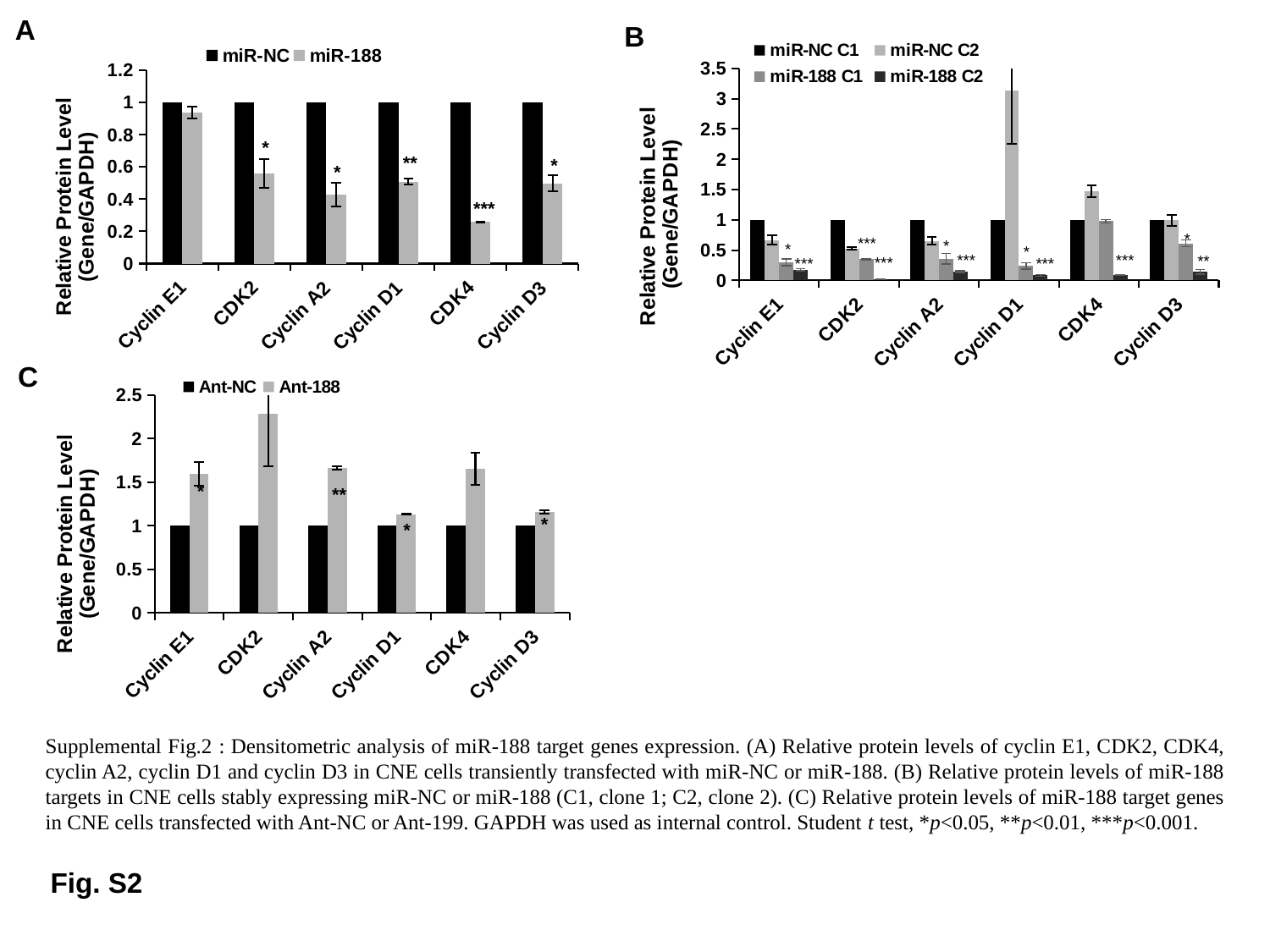

### Chart
| Category | miR-NC | miR-188 |
|---|---|---|
| Cyclin E1 | 1.0 | 0.9370920790392342 |
| CDK2 | 1.0 | 0.5586114494787208 |
| Cyclin A2 | 1.0 | 0.4276474352349233 |
| Cyclin D1 | 1.0 | 0.5088251162368658 |
| CDK4 | 1.0 | 0.2572078816221778 |
| Cyclin D3 | 1.0 | 0.49693495168270135 | *
 **
 *
 *
 ***
A
B
### Chart
| Category | miR-NC C1 | miR-NC C2 | miR-188 C1 | miR-188 C2 |
|---|---|---|---|---|
| Cyclin E1 | 1.0 | 0.6675631714822843 | 0.2980735226535351 | 0.17517696442970218 |
| CDK2 | 1.0 | 0.5226184542084172 | 0.35033103459035725 | 0.019393462039757586 |
| Cyclin A2 | 1.0 | 0.6484906499715734 | 0.3591800258867011 | 0.14640746039612057 |
| Cyclin D1 | 1.0 | 3.1391843759535014 | 0.23735347416786948 | 0.08194525331012151 |
| CDK4 | 1.0 | 1.475161195565071 | 0.976810240903961 | 0.0910795774741225 |
| Cyclin D3 | 1.0 | 0.9893046726364607 | 0.6100790589231239 | 0.14345337789821724 | *
 ***
 *
 *
 *
 ***
 ***
 **
 ***
 ***
 ***
C
### Chart
| Category | Ant-NC | Ant-188 |
|---|---|---|
| Cyclin E1 | 1.0 | 1.596045001122345 |
| CDK2 | 1.0 | 2.287761011758527 |
| Cyclin A2 | 1.0 | 1.6623710361397863 |
| Cyclin D1 | 1.0 | 1.1341574590718344 |
| CDK4 | 1.0 | 1.6553642200069458 |
| Cyclin D3 | 1.0 | 1.1596263287801543 | *
 **
 *
 *
Supplemental Fig.2 : Densitometric analysis of miR-188 target genes expression. (A) Relative protein levels of cyclin E1, CDK2, CDK4, cyclin A2, cyclin D1 and cyclin D3 in CNE cells transiently transfected with miR-NC or miR-188. (B) Relative protein levels of miR-188 targets in CNE cells stably expressing miR-NC or miR-188 (C1, clone 1; C2, clone 2). (C) Relative protein levels of miR-188 target genes in CNE cells transfected with Ant-NC or Ant-199. GAPDH was used as internal control. Student t test, *p<0.05, **p<0.01, ***p<0.001.
Fig. S2
