# Supplementary material for: MicroRNA-188 suppresses G1/S transition by targeting multiple cyclin/CDK complexes
Source: Cell Commun Signal. 2014 Oct 11;12:66. doi: 10.1186/s12964-014-0066-6 (PMC4200121; doi:10.1186/s12964-014-0066-6)
Supplement: Additional file 4: Figure S3. — Stable expression of miR-188 inhibits G1/S transition and Rb phosphorylation. (A) Flow Cytometry analysis of CNE cells stably expressing miR-NC or miR-188 released from hydroxyurea for 6 h. (B) Relative levels of Rb phosphorylation were quantified by densitometric analysis. Total Rb was used as internal control. Student t test, ** p < 0.01, ***p < 0.001. (C) Immunoblot analysis of phosphor-Rb S811, phosphor-Rb S780, total Rb and GAPDH in CNE cells stably expressing miR-NC or miR-188. (D) Relative levels of Rb phosphorylation in (C) were quantified by densitometric analysis, total Rb was used as internal control. Student t test, *p < 0.05, ** p < 0.01. [file 12964_2014_66_MOESM4_ESM.pptx]

## Slide 1
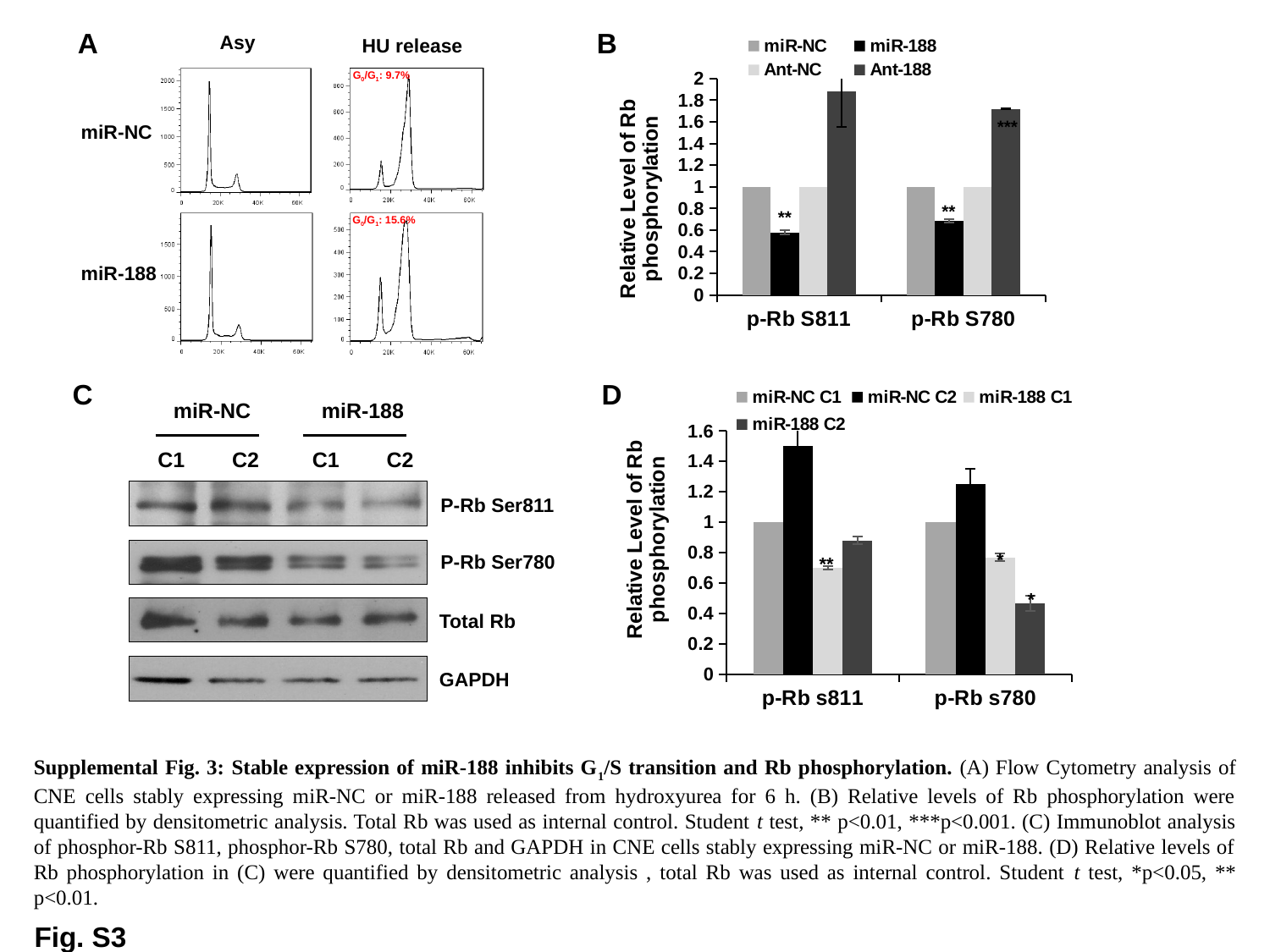

A B
### Chart
| Category | miR-NC | miR-188 | Ant-NC | Ant-188 |
|---|---|---|---|---|
| p-Rb S811 | 1.0 | 0.5780182436864486 | 1.0 | 1.8783406180726199 |
| p-Rb S780 | 1.0 | 0.6844531287277686 | 1.0 | 1.7199198541067102 | ***
 **
 **
Asy
HU release
G0/G1: 9.7%
miR-NC
miR-188
G0/G1: 15.6%
### Chart
| Category | miR-NC C1 | miR-NC C2 | miR-188 C1 | miR-188 C2 |
|---|---|---|---|---|
| p-Rb s811 | 1.0 | 1.5011790790732271 | 0.69917577457983 | 0.8803802079158634 |
| p-Rb s780 | 1.0 | 1.252573138349768 | 0.7685628612025801 | 0.4653518102418383 | *
 **
 *
C
D
 miR-NC miR-188
C1 C2 C1 C2
P-Rb Ser811
P-Rb Ser780
Total Rb
GAPDH
Supplemental Fig. 3: Stable expression of miR-188 inhibits G1/S transition and Rb phosphorylation. (A) Flow Cytometry analysis of CNE cells stably expressing miR-NC or miR-188 released from hydroxyurea for 6 h. (B) Relative levels of Rb phosphorylation were quantified by densitometric analysis. Total Rb was used as internal control. Student t test, ** p<0.01, ***p<0.001. (C) Immunoblot analysis of phosphor-Rb S811, phosphor-Rb S780, total Rb and GAPDH in CNE cells stably expressing miR-NC or miR-188. (D) Relative levels of Rb phosphorylation in (C) were quantified by densitometric analysis , total Rb was used as internal control. Student t test, *p<0.05, ** p<0.01.
Fig. S3
