# Supplementary material for: MicroRNA-188 suppresses G1/S transition by targeting multiple cyclin/CDK complexes
Source: Cell Commun Signal. 2014 Oct 11;12:66. doi: 10.1186/s12964-014-0066-6 (PMC4200121; doi:10.1186/s12964-014-0066-6)
Supplement: Additional file 5: Table S2 and Table S3. — Primers used for qRT-PCR (Table S2) and luciferase activity assay (Table S3). [file 12964_2014_66_MOESM5_ESM.docx]

**Table S2. Primers used for qRT-PCR.**

| **Genes** | **Forward** | **Reverse** |
| --- | --- | --- |
| CCND1 | CAATGACCCCGCACGATTTC | CATGGAGGGCGGATTGGAA |
| CCND3 | TACCCGCCATCCATGATCG | AGGCAGTCCACTTCAGTGC |
| CCNE2 | TCAAGACGAAGTAGCCGTTTAC | TGACATCCTGGGTAGTTTTCCTC |
| b-MYB | CCGGAGCAGAGGGATAGCA | CAGTGCGGTTAGGGAAGTGG |
| DHFR | CAAAGACTCAGGGGTTACCTC | ATTCCACCCAGCTAACCGAT |
| CCNE1 | ACTCAACGTGCAAGCCTCG | GCTCAAGAAAGTGCTGATCCC |
| CCNA2 | GGATGGTAGTTTTGAGTCACCAC | CACGAGGATAGCTCTCATACTGT |
| CDK2 | TGCACTACGACCCTAACAAGC | ACCTGAGTCCAAATAGCCCAA |
| CDK4 | CCTACCTTTATATTTGGGGTCCT | GGCCCTGTAATTTAACCAGT |

**Table S3. Primers used for luciferase activity assay.**

| **Genes** | **Forward primer** | **Reverse primer** |
| --- | --- | --- |
| CDK4-UTR-WT | AGCGAGCTCTTTCCCTTCTGGACACTGAGA | GCTCTAGATCCAAATCGCACAATGGCAAA |
| CDK4-UTR-Mu | ATGCTGGATGTTCCCTCTTGTCC | AGTGTAGAGAAATGGGAAGGAGA |
| CDK2-UTR-Mu | ATCGAGCTCCAGCCCTAATCTCACCCTCTC | GCTCTAGAACGGCAAATCTAACGTGTAGGAA |
| CDK2-UTR-WT | AATCGCGGGAACCAGTGAAAATG | CTCGCGAGTATTCCCAGAGTTGG |
| CCNA2-UTR-WT | AGCGAGCTCATTTCTTAGTTTTCCAGTAGG | GCTCTAGAAATGTATACATATACTCAACACT |
| CCNA2-UTR-Mu | GCTACTCTTTTTGTTTATAGTCATG | GTATTTCATTAGAGATCCATCTG |
| CCNE1-UTR-WT | ATCGAGCTCAAGTATTTCTGTGGATGGCATCA | GCTCTAGAGTCAACTGATAATGTGGAGAGGG |
| CCNE1-UTR-Mu | GCGCTCTCCCACAACAACAAAAG | TAGGTCAAGTAGCACCTTCCATA |
| CCND1-UTR-WT | ATCGAGCTCAGGAAGAGGAGGAGGAGGAG | GCTCTAGACGCCAAACAGGCTGAATCAAT |
| CCND1-UTR-Mu | GCGTAAGCTTCATTCTCCTTGTTG | AACTGGTATCAAAATGCTCCGGA |
| CCND3-UTR-WT | AGCGAGCTCCCCTCTGGAGTGGCCACTAA | GGACTAGTGCCTTCAGCAGCAAAGCTGTC |
| CCND3-UTR-Mu | TCGCGTACTGGTCCTACCCATCC | GGGCTTTGTGAAGGGGGAACAG |
